# Impact of PARP Inhibitors on Health-related Quality of Life in Patients with Metastatic Castration-resistant Prostate Cancer: A Systematic Review and Meta-analysis

**DOI:** 10.1016/j.euros.2025.08.008

**Published:** 2025-09-08

**Authors:** Navid Roessler, Marcin Miszczyk, Akihiro Matsukawa, Alessandro Dematteis, Ahmed R. Alfarhan, Angelo Cormio, Abdulrahman S. Alqahtani, Pawel Rajwa, Victor M. Schuettfort, Malte W. Vetterlein, Timo F.W. Soeterik, Tamás Fazekas, Margit Fisch, Michael S. Leapman, Pierre I. Karakiewicz, Shahrokh F. Shariat

**Affiliations:** a Department of Urology, Comprehensive Cancer Center, Medical University of Vienna, Vienna, Austria; b Department of Urology, University Medical Center Hamburg-Eppendorf, Hamburg, Germany; c Collegium Medicum, Faculty of Medicine, WSB University, Dąbrowa Górnicza, Poland; d Department of Urology, Jikei University School of Medicine, Tokyo, Japan; e Department of Urology, Città della Salute e della Scienza, University of Torino School of Medicine, Torino, Italy; f Department of Urology, Prince Saud Bin Jalawi Hospital, Al Ahsa Health Cluster, Al Ahsa, Saudia Arabia; g Department of Urology, Azienda Ospedaliero-Universitaria Ospedali Riuniti Di Ancona, Università Politecnica Delle Marche, Ancona, Italy; h Department of Urology and Renal Transplantation, Policlinico Riuniti di Foggia, University of Foggia, Foggia, Italy; i Department of Urology, Second Health Cluster Riyadh, Riyadh, Saudia Arabia; j Department of Urology, Centre of Postgraduate Medical Education, Warsaw, Poland; k Division of Surgery and Interventional Sciences, University College London, London, UK; l Department of Radiation Oncology, University Medical Center Utrecht, Utrecht, The Netherlands; m Centre for Translational Medicine, Semmelweis University, Budapest, Hungary; n Department of Urology, Semmelweis University, Budapest, Hungary; o Department of Urology, Yale University, New Haven, CT, USA; p Cancer Prognostics and Health Outcomes Unit, Division of Urology, University of Montréal Health Center, Montréal, Canada; q Department of Urology, University of Texas Southwestern, Dallas, TX, USA; r Hourani Center for Applied Scientific Research, Al-Ahliyya Amman University, Amman, Jordan; s Karl Landsteiner Institute of Urology and Andrology, Vienna, Austria; t Research Center for Evidence Medicine, Urology Department, Tabriz University of Medical Sciences, Tabriz, Iran

**Keywords:** Prostate cancer, Health-related quality of life, Functional Assessment of Cancer Therapy-Prostate, EQ-5D-5L questionnaire, Metastatic cancer

## Abstract

**Background and objective:**

PARP inhibitor (PARPi) agents, alone or in combination with antiandrogens, are considered a standard of care for patients with metastatic castration-resistant prostate cancer (mCRPC). This meta-analysis evaluates the impact of PARPi agents on health-related quality of life (HRQoL) using data from prospective trials.

**Methods:**

In this prospectively registered systematic review and meta-analysis (PROSPERO: CRD420251011282), we searched the MEDLINE, Embase, and Web of Science databases in March 2025 for prospective trials assessing the impact of PARPi on patient HRQoL in mCRPC. Results for the mean differences (MD) in Functional Assessment of Cancer Therapy-Prostate (FACT-P) total scores at early and late time points were pooled using a random-effects model.

**Key findings and limitations:**

Reports for seven prospective trials (2861 patients) were included. The PROfound trial (*n* = 387) demonstrated a clinically meaningful improvement in global HRQoL (MD from baseline: 6.7, 95% confidence interval [CI] 1.5–12), while the remaining six studies (*n* = 2474) did not show a clinically meaningful impact individually. In pooled analysis for the PROpel, MAGNITUDE, and NCT01972217 randomised controlled trials (*n* = 911), no clinically meaningful differences in the change in FACT-P total score were observed at early (weeks 8–9: MD −1.4, 95%CI −3.0 to 0.2) or late (weeks 96–101: MD −2.1, 95%CI −8.9 to 4.8) time points. Six studies were rated as having some/moderate concerns regarding bias, and one was considered at high risk of bias because of baseline stratification by symptom status.

**Conclusions and clinical implications:**

We observed no statistically or clinically meaningful deterioration in HRQoL with PARPi treatment in patients with mCRPC, with one study suggesting a potential benefit. Predefined thresholds may not capture the individual and complex nature of HRQoL in the palliative mCRPC setting, where even modest changes can have important clinical and personal significance, which underscores the importance of nuanced interpretation of HRQoL data in guiding treatment decisions.

**Patient summary:**

We did not find evidence that drugs called PARP inhibitors have a negative effect on health-related quality of life (HRQoL) for men with advanced prostate cancer. However, the current tools used to measure HRQoL may not fully reflect the individual and complex experiences of patients receiving palliative treatment for their cancer.

## Take Home Message

We found no statistically or clinically meaningful deterioration in health-related quality of life (HRQoL) with PARP inhibitor (PARPi) in patients with metastatic castration-resistant prostate cancer (mCRPC), and one study suggested a potential benefit. These findings support the favourable balance between the efficacy and tolerability of PARPi treatment in mCRPC management, although current HRQoL assessment tools may underestimate subtle but clinically relevant patient experiences in the palliative setting.

## 1 Introduction

Metastatic castration-resistant prostate cancer (mCRPC) is an incurable systemic disease marked by progression despite effective androgen suppression and represents a clinically challenging terminal stage of prostate cancer (PC). Therapeutic options for these men are limited, with median overall survival estimated at approximately 3 yr in clinical trial settings [1,2] and <2 yr in real-world settings [3]. PARP inhibitor (PARPi) agents have recently emerged as a novel class of targeted therapies for mCRPC in patients with homologous recombination repair (HRR) gene mutations, including *BRCA* [[4], [5], [6], [7], [8]]. Given the profound impact of mCRPC and its treatments on patient physical, emotional, and social wellbeing, health-related quality of life (HRQoL) is an important factor for patients and should therefore receive significant attention in patient counselling and treatment decision-making [9].

Patient preferences and personal goals should guide treatment choice [10,11], with patients frequently willing to make trade-offs between survival and HRQoL [12]. An understanding of these preferences is essential for providing a comprehensive approach to cancer management in each disease stage and ensuring that treatment choices align with patient priorities. Therefore, our aim for this systematic review and meta-analysis was to provide a comprehensive understanding of the HRQoL impact of PARPi therapy in patients with mCRPC.

## 2 Methods

This systematic review was registered with the International Prospective Register of Systematic Reviews (PROSPERO: CRD420251011282) and conducted in accordance with the Preferred Reporting Items for Systematic Reviews and Meta-analyses (PRISMA) statement (Supplementary Fig. 1) and the AMSTAR-2 checklist (Supplementary Table 4) [13,14].

### 2.1 HRQoL definitions

HRQoL was assessed using standardised tools. Functional Assessment of Cancer Therapy-Prostate (FACT-P) is a PC-specific HRQoL questionnaire [15]. The total score ranges from 0 to 156, with higher scores indicating better HRQoL. Clinically meaningful changes are defined as a score change of ≥6 or ≥ 10 [16]. The EuroQol 5-dimension 5-level (EQ-5D-5L) tool is a self-assessment questionnaire assessing health status across five dimensions: mobility, self-care, usual activities, pain/discomfort, and anxiety/depression, with five response options [17]. It also includes a visual analogue scale (VAS) for rating current health from 0 (worst) to 100 (best). The responses generate a unique health state that is converted into a summary score (health utility index [HUI]) ranging from 0 (death) to 1 (best health).

### 2.2 Study selection

The research question and inclusion criteria were defined using the PICOS (Population, Intervention, Comparison, Outcome, and Study design) framework (Supplementary Fig. 2). We searched the MEDLINE (via PubMed), Embase, and Web of Science Core Collection databases for reports on trials that assessed HRQoL in patients with mCRPC treated with PARPi. We included all prospective trial reports, including subset and post hoc analyses of the study groups, that evaluated HRQoL assessed using the EQ-5D-5L or FACT-P questionnaire [15,17]. We excluded manuscripts not in English, studies not providing original data, editorials, and review articles. Furthermore, for cases in which multiple reports involved overlapping patient cohorts, the report with the largest sample size, relevant endpoint, and most recent data was selected for inclusion in the analysis.

The search strategy was performed in March 2025. The detailed search strategy is provided in Supplementary Table 1. Reports were merged and deduplicated using EndNoteX9 (Clarivate, London, UK). Screening by title and abstract was conducted independently by two authors. Relevant full-text reports were then retrieved and screened independently by two authors. Backward citation searching was performed to identify potentially relevant additional records. At each step of the review, conflicts were resolved via consensus among co-authors.

### 2.3 Data extraction

Two authors independently extracted details including the first author’s name, publication year, clinical trial phase, patient characteristics (age, sample size, and performance status), tumour characteristics (genomic mutations and metastatic sites), HRQoL data, and baseline pain severity assessed via the Brief Pain Inventory-Short Form (BPI-SF) questionnaire [18]. All conflicts that arose during the data extraction process were resolved via discussion between the two authors.

Changes in HRQoL were assessed at different time points. Early HRQoL was evaluated between weeks 8 and 9, while late HRQoL was assessed between weeks 96 and 101, representing the latest time points available. The analysis focused on the mean difference (MD) from baseline in FACT-P total score for each respective period to capture changes in HRQoL over time. When appropriate outcome measures were not reported directly, curves reporting the least-square mean change in FACT-P score from baseline were digitised using WebPlotDigitizer v4.6.0 to extract the necessary data [19].

### 2.4 Risk of bias

The risk of bias (RoB) for each study was evaluated independently by two authors using the Cochrane Collaboration’s RoB version 2.0 (RoB2) tool for randomised controlled trials (RCTs) [20], and the Risk of Bias in Nonrandomised Studies of Interventions (ROBINS-I) tool for nonrandomised studies [21].

### 2.5 Statistical analysis

The meta-analysis was performed using the random-effects inverse variance method with a restricted maximum-likelihood estimator, applying log-transformed mean changes from baseline (treatment arm and control arm) with the corresponding standard error (SE) values as input. Heterogeneity was evaluated and further explored via leave-one-out sensitivity analyses (Supplementary Fig. 3). In cases of significant heterogeneity, differences between studies were assessed qualitatively. The outcomes are presented as forest plots of pooled MD values with corresponding 95% confidence interval (CI). All tests were two-sided.

## 3 Results

Seven studies involving 2861 patients with mCRPC were included [[22], [23], [24], [25], [26], [27], [28]]. Five studies were subsequent analyses of RCTs [22,23,25,27,28], while two were based on prospective single-arm trials [24,26].

### 3.1 RoB assessment

The RoB judgments for each domain for each study included are summarised in Supplementary Table 2. According to RoB2 for RCTs and ROBINS-I for nonrandomised studies, there were some/moderate concerns for six studies. One study was considered to be at high RoB [22] because of separation of the patients according to their baseline BPI-SF score, introducing potential bias to HRQoL assessment.

### 3.2 Study characteristics

Three studies (*n* = 462) reported results from phase 2 trials [6,29,30], while the other four studies (*n* = 2399) reported results from phase 3 trials [4,5,31,32]. In the PROpel (*n* = 796) and MAGNITUDE (*n* = 423) trials, PARPi agents were given in first-line mCRPC setting [4,31]. The PROpel trial and trial NCT01972217 (*n* = 142) compared the combination of olaparib and abiraterone to placebo and abiraterone [29,31]. PROfound (*n* = 387) assessed olaparib as monotherapy in comparison to enzalutamide or abiraterone [5]. The MAGNITUDE trial assessed niraparib in combination with abiraterone versus placebo and abiraterone [4], while GALAHAD (*n* = 223) assessed niraparib as monotherapy without a comparator arm [30]. Lastly, the TALAPRO-1 trial (*n* = 127) evaluated talazoparib as monotherapy without a control group [6], and TALAPRO-2 (*n* = 805) assessed talazoparib in combination with enzalutamide versus placebo and enzalutamide [32]. Four trials included patients with Eastern Cooperative Oncology Group (ECOG) performance status ≤2 [[24], [25], [26], [27]], while three trials included only patients with ECOG performance status ≤1 [22,23,28]. Six studies (*n* = 2474) reported baseline BPI-SF scores, with the majority of patients having mild pain [[22], [23], [24], [25], [26],^28^]. One study (*n* = 796) reported outcomes stratified by baseline BPI-SF score and opioid use [22]. Table 1 summarises the characteristics of the studies included. Further questionnaire assessments are detailed in Supplementary Table 3.

**Table 1:** Demographics and trial characteristics for the studies included in the review

**Trial and any baseline**	**Pts,**	**Treatment arm**	**Pts,**	**Median**	**ECOG PS,**	**Site of metastasis, *n* (%)**	**Baseline scores**	
**stratification**	***n* (%)**		***n* (%)**	**age, yr**	***n* (%)**		**BPI-SF question 3**	**FACT-P**
				**(range)**				**total score**
PROpel (2018–2020) Clarke [22]	796							
Asymptomatic or mildly symptomatic	560 (70)	Olaparib + Abi	266 (47)	69 (45–91)	0: 215 (81) 1: 51 (19)	Bone 223 (84); DLN 89 (34); LLN 51 (19); lung 27 (10); liver 6 (2.3)	0: 133 (50%) >0–<4: 133 (50%)	NR
		Placebo + Abi	294 (53)	70 (48–87)	0: 225 (76) 1: 69 (24)	Bone 246 (84); DLN 91 (31); LLN 66 (22); lung 32 (11); liver 11 (3.7)	0: 136 (46%) >0–<4: 158 (54%)	NR
Symptomatic	183 (23)	Olaparib + Abi	103 (56)	69 (43–89)	0: 53 (52) 1: 50 (49)	Bone 99 (96); DLN 34 (33); LLN 24 (23); lung 9 (8.7); liver 7 (6.8)	>0–<4: 18 (18%) 4–<6: 53 (52%) ≥6: 32 (31%)	NR
		Placebo + Abi	80 (44)	71 (46–88)	0: 37 (46) 1: 43 (54)	Bone 73 (91); DLN 20 (25); LLN 19 (24); lung 9 (11); liver 5 (6.3)	0: 1 (1.3%) >0–<4: 15 (19%) 4–<6: 36 (45%) ≥6: 28 (35%)	NR
MAGNITUDE (2019–2023) Rathkopf [23]	423	Niraparib + Abi	212 (50)	69 (45–100)	0: 130 (61) 1: 82 (39)	Bone 183 (86); LN 113 (53); visceral 51 (24); other: 6 (3)	0 (0–2) a	120 (104–129) a
		Placebo + Abi	211 (50)	69 (43–88)	0: 146 (69) 1: 65 (31)	Bone 170 (81); LN 95 (45); visceral 39 (19); other 15 (7.1)	1 (0–2) a	118 (105–129) a
TALAPRO-1 (2017–2020) Saad [24]	97	Talazoparib	97 (100)	69 (63–73) a	0: 41 (42) 1: 49 (51) 2: 7 (7.2)	Visceral 32 (33)	<4: 45 (46%) ≥4: 38 (39%)	NR
TALAPRO-2 (2019–2020) Matsubara [28]	793	Talazoparib + Enza	395 (50)	71 (66–76) a	NR	NR	1.7 (1.4–1.9) b	NR
		Placebo + Enza	398 (50)	71 (65–76)	NR	NR	1.8 (1.5–2) b	NR
NCT01972217 (2014–2015) Saad [25]	142	Olaparib + Abi	71 (50)	70 (65–75) a	0: 34 (48) 1: 36 (51) 2: 1 (1)	Bone 33 (46); ST 8 (11); bone and ST 30 (42)	3.5 (2.7) c	101 (19) c
		Placebo + Abi	71 (50)	67 (62–74) a	0:38 (54) 1:30 (42) 2: 1 (1)	Bone 33 (46); ST 11 (15); bone and ST 27 (38)	3.3 (2.9) c	106 (19) c
GALAHAD (2016–2020) Smith [30]	223							
*BRCA* mutation	142 (64)	Niraparib	142 (64)	67 (46–86)	0: 48 (34) 1: 78 (55) 2: 16 (11)	NR	3.2 (2.3) c	97 (22) c
Other HRR mutation	81(36)	Niraparib	81 (36)	70 (52–88)	0: 18 (22) 1: 47 (58) 2: 16 (20)	NR	2.6 (2.3) c	101 (22) c
PROfound (2017–2019) Thiery-Vuillemin [27]	387	Olaparib	256 (66)	69 (63–74) a	0: 131 (51) 1: 112 (44) 2: 13 (5)	Bone 86 (34); visceral (lung or liver) 68 (27); other 88 (34)	NR	107 (104–110) b
		Enza or Abi	131 (34)	69 (64–73) a	0: 55 (42) 1: 71 (54) 2: 4 (3)	Bone 38 (29); visceral (lung or liver) 44 (34); other 41 (31)	NR	108 (104–112) b

Abi = abiraterone; BPI-SF = Brief Pain Inventory-Short Form; DLN = distant LN; ECOG PS = Eastern Cooperative Oncology Group performance status; Enza = enzalutamide; FACT-P = Functional Assessment of Cancer Therapy-Prostate; HRR = homologous recombination repair; LN = lymph nodes; LLN = locoregional LN; Pts = patients; NR = not reported; ST = soft tissue.

a Median (interquartile range).

b Mean (95% confidence interval).

c Mean (standard deviation).

### 3.3 FACT-P results for HRQoL

Five studies (*n* = 1971) evaluated the impact of PARPi treatment on HRQoL using the FACT-P questionnaire [22,23,[25], [26], [27]]. Of these, three studies (*n* = 1325) reported overall MDs in FACT-P total scores between treatment and placebo arms [22,25,27], with one study (*n* = 387) demonstrating a clinically meaningful improvement in HRQoL assessed via the FACT-P total score [27].

In the analysis of the phase 3 PROpel trial (*n* = 796), the least-squares mean change from baseline in FACT-P total score was −5.2 points (SE 1.1) with olaparib versus −5.7 points (SE 1.1) with placebo in the asymptomatic/mildly symptomatic group; the difference between arms was 0.4 points (95% CI −2.2 to 3.0). Among symptomatic patients, the change was −4.4 points (SE 2.3) for olaparib and 1.9 points (SE 2.5) for placebo, with a difference of −6.3 (95% CI −12 to −0.4). While the HRQoL scores with olaparib were numerically lower in the symptomatic group, the difference did not meet the predefined threshold for a clinically meaningful change (≥10 points) [22].

In the analysis of the phase 3 MAGNITUDE trial (*n* = 423), baseline FACT-P scores were comparable between the treatment arms. No clinically meaningful HRQoL differences were observed at early (cycle 2: −1.8 points, 95% CI −3.9 to 0.4 vs 1.26 points, 95% CI −0.9 to 3.4) or late (cycle 28: −5.0 points, 95% CI −8.1 to −1.9 vs −2.1 points, 95% CI −5.5 to 1.4) time points. Both groups showed slight overall declines and small, nonsignificant differences favouring niraparib in the group with BRCA mutations (−1.0 points, 95% CI −5.2 to 3.3) versus the overall group with HRR mutations (−5.0 points, 95% CI −8.1 to −1.9), none of which reached the predefined threshold for clinical significance (≥10-point change in FACT-P total score) [23]. In an analysis of the phase 2 NCT01972217 trial (*n* = 142), baseline FACT-P scores were comparable between the treatment arms. The least-squares mean change from baseline in FACT-P total scores was −0.6 points (95% CI −4.2 to 3.0) in the olaparib group and −2.1 points (95% CI −6.1 to 2.0) in the placebo group, resulting in a difference of 1.5 points (95% CI −4.0 to 6.9), which did not meet the study-defined threshold for a clinically meaningful change (≥6 points) [25].

According to analysis of the phase 2 GALAHAD trial (*n* = 223), baseline FACT-P total scores were similar between the *BRCA* and non-*BRCA* HRR mutation groups. In the *BRCA* mutation group, HRQoL improved by cycle 3 (6.0 points, 95% CI 2.8–9.3) and remained above baseline up to cycle 10 (2.8 points, 95% CI −2.0 to 7.6). By contrast, no early improvement was observed in the non-*BRCA* HRR mutation group (−0.1 points, 95% CI −4.7 to 4.6), with scores declining by cycle 10 (−5.1 points, 95% CI −15 to 5.1). None of these changes met the predefined threshold for clinical significance (≥10-point change in FACT-P total score) [26].

Finally, in a subsequent analysis of the phase 3 PROfound trial (*n* = 387), baseline FACT-P total scores were comparable between the olaparib and control arms. The overall difference in mean change from baseline in FACT-P total score between the olaparib and placebo groups was 6.7 points (95% CI 1.5–12), which met the predefined threshold for clinical meaningfulness (≥6-point change). In the subgroup with *BRCA* or *ATM* mutations (*n* = 245), the olaparib arm (*n* = 162) showed improved HRQoL from week 4 onwards in comparison to the control arm, with differences maintained up to week 32 [27].

### 3.4 Meta-analysis of FACT-P scores

Three studies reported the mean change from baseline FACT-P total score at early (weeks 8–9) and late (weeks 96–101) time points for the treatment arm (*n* = 443) and control arm (*n* = 468) [22,23,25].

**Fig. 1:**
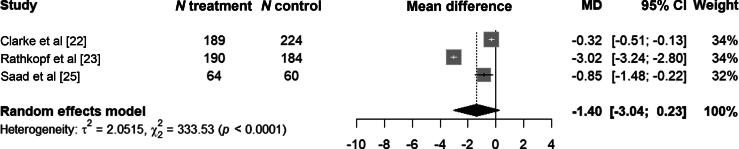
Forest plot of the pooled mean difference (MD) in Functional Assessment of Cancer Therapy-Prostate (FACT-P) total scores between the treatment and control arms in the early time period. CI = confidence interval.

The pooled MD between the treatment and control arms for the change in FACT-P total score during the early period was −1.4 points (95% CI −3.0 to 0.2; Fig. 1). There was evidence of significant heterogeneity, with leave-one-out analysis identifying one study as a major contributor to this heterogeneity [23] (Supplementary Fig. 6). After excluding the study identified as the main source of heterogeneity, the pooled MD for the change in FACT-P total score during the early period was −0.5 points (95%CI −1.0 to −0.01; Supplementary Fig. 4).

**Fig. 2:**
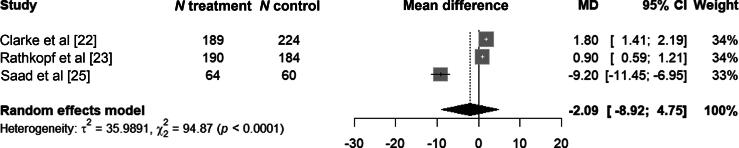
Forest plot of the pooled mean difference (MD) in Functional Assessment of Cancer Therapy-Prostate (FACT-P) total scores between the treatment and control arms in the late time period. CI = confidence interval.

The pooled MD between the treatment and control arms for the change in FACT-P total score during the late period was −2.1 points (95% CI −8.9 to 4.8; Fig. 2). There was evidence of significant heterogeneity, with leave-one-out analysis identifying one study as a major contributor to this heterogeneity [25] (Supplementary Fig. 6). After excluding the study identified as the main source of heterogeneity, the pooled MD in FACT-P total score change during the late period was 1.3 (95% CI 0.5–2.2; Supplementary Fig. 4).

The heterogeneity observed probably reflects variations in study design, the characteristics of the patient populations, the assessment time points, and the individual and subjective nature of HRQoL perception and reporting. When assessing clinically meaningful changes in FACT-P total scores using a threshold of ≥10 points, none of the pooled results indicated a clinically significant deterioration. Applying the lower threshold of ≥6 points identified a clinically meaningful decline in FACT-P in one study during the late period (MD −9.2 points, 95%CI −11 to −7.0) [25], although this finding was not reflected in the overall pooled analyses for either early or late time points (Fig. 1, Fig. 2).

### 3.5 EQ-5D-5L results for HRQoL

Four studies (*n* = 1455) reported HRQoL outcomes using the EQ-5D-5L questionnaire [[23], [24], [25],^28^].

In an analysis of the phase 2 TALAPRO-1 trial (*n* = 97), the mean changes from baseline in the overall population were 0.05 (95% CI 0.01–0.1) for the EQ-5D-5L HUI and 5.4 points (95% CI 2.7–8.2) for the VAS. Improvements were noted in mobility (−0.2, 95% CI −0.3 to −0.003) and pain/discomfort (−0.3, 95% CI −0.5 to −0.2) dimensions for all patients, while usual activities improved in the *BRCA* mutation subgroup (−0.2, 95% CI −0.4 to −0.04) [24].

Analysis of the phase 3 MAGNITUDE trial (*n* = 423) revealed that baseline median EQ-5D-5L HUI and VAS scores were comparable between the niraparib and control arms in both the overall population and the *BRCA* mutation subgroup. Throughout treatment, only minor differences were observed in both VAS (<5 points) and HUI scores, without evidence of meaningful divergence between the groups [23].

In an analysis of the phase 3 TALAPRO-2 trial (*n* = 793), no significant difference in general health status was observed between treatment arms, as measured via EQ-5D-5L HUI scores (*p* = 0.37) and VAS scores (MD −0.6 points, 95% CI −2.5 to 1.3; *p* = 0.55) [28].

Finally, analysis of the phase 2 NCT01972217 trial (*n* = 142) revealed comparable baseline EQ-5D-5L VAS scores between the olaparib and control arms. Pain/discomfort improvements were similar between the groups up to week 48, after which a greater proportion of patients receiving olaparib reported sustained improvement relative to patients in the control arm [25].

## 4 Discussion

Across the studies included, we did not observe a clinically meaningful negative impact of PARPi on HRQoL, with one study even demonstrating a clinically meaningful benefit. This suggests that PARPi agents do not negatively affect HRQoL in comparison to placebo. Thus, the negative effects of PARPi therapy seem tolerable to patients and are not worse than those of placebo. This preservation of HRQoL is particularly relevant given the known survival benefits of PARPi therapy [4,5,29,31,32], with the most pronounced effects observed for patients harbouring *BRCA* mutations [33].

The adverse events associated with PARPi treatment most frequently reported, including anaemia, thrombocytopenia, neutropenia, fatigue, and nausea [4,6,30,31,34], are generally manageable and tend to occur predominantly within the first 6 mo of therapy [35]. Interestingly, our meta-analysis revealed no meaningful difference between early and late mean changes in the FACT-P total score, suggesting that adverse events may not substantially impair HRQoL over the course of treatment, or that they might be balanced by the improvement in disease control. However, interpretation is inherently complex despite the use of established instruments [36], particularly in this palliative context. This underscores the dual nature of therapeutic benefit in the mCRPC setting, where both an improvement in HRQoL via symptom relief, and maintenance of HRQoL despite treatment-related toxicities may represent clinically meaningful outcomes.

HRR mutations, present in approximately a quarter of patients with advanced PC, are associated with poor prognosis and resistance to standard therapies, making these patients eligible for PARPi therapy [[37], [38], [39], [40]]. While PARPi agents have a clinically meaningful benefit in patients with *BRCA* mutations [4,41], HRQoL improvements are generally modest. Analyses for the MAGNITUDE and PROfound trials demonstrated that PARPi agents provide improvements in HRQoL for patients with *BRCA* mutations [23,27]; the GALAHAD trial confirmed this with a larger effect [26]. Although the effect was not clinically meaningful according to the predefined differences in points, these studies demonstrated a HRQoL benefit of PARPi therapy in patients with *BRCA*-mutated mCRPC, with no clear disadvantages observed, supporting their continued use in this subgroup of patients. EQ-5D-5L results similarly showed modest improvements in selected domains and no significant decline in overall HRQoL. Conversely, recent data from the MAGNITUDE trial showed no significant overall survival benefit of niraparib plus abiraterone over placebo in either the HRR or *BRCA* mutation subgroups, despite earlier improvements in progression-related endpoints [42].

Alternative treatments such as cabazitaxel and lutetium-177-PSMA-617 are established options for patients with mCRPC and serve as relevant comparators when evaluating the clinical and HRQoL impact of PARPi. In the phase 2 TheraP trial, lutetium-177-PSMA-617 showed superior efficacy and a more favourable safety profile in comparison to cabazitaxel, with higher prostate-specific antigen response rates, longer progression-free survival, and fewer grade 3–4 adverse events [43]. This treatment was also associated with clinically meaningful improvements in HRQoL, including reduced fatigue, better social functioning, and improvements in insomnia, as well as longer deterioration-free survival in global health status [43]. Despite their known toxicity profiles, both cabazitaxel and lutetium-177-PSMA-617 have shown the potential to preserve or even improve HRQoL in selected patient populations [[44], [45], [46]]. These findings emphasise the relevance of HRQoL assessment when considering PARPi for patients with advanced disease, for whom treatment goals extend beyond survival.

While none of the studies included demonstrated clinically meaningful changes in HRQoL during the early period, one study reported a clinically meaningful deterioration in the late period when applying the more sensitive threshold for the FACT-P total score of ≥6 points [25]. However, significant heterogeneity in the pooled analyses limits the interpretability of these findings and highlights the challenges in drawing definitive conclusions from the data available. In the early-phase assessment, heterogeneity was primarily driven by the MAGNITUDE trial, which included only patients with HRR-mutated mCRPC and a higher symptom burden at baseline [23]. These patients may experience different progression of HRQoL deterioration because of more aggressive disease biology with greater initial impairment. Late-phase heterogeneity was mainly driven by the phase 2 NCT01972217 trial, which included a more clinically heterogeneous patient cohort with varied prior treatments and symptom severity, and less standardised follow-up schedules, which probably contribute to greater variability in long-term HRQoL outcomes [25]. Nevertheless, even minor changes in FACT-P scores warrant consideration for patients with mCRPC given the palliative context of this disease stage. As physical wellbeing remained largely stable, the HRQoL decline observed for symptomatic patients receiving olaparib may reflect their worse baseline prognosis, characterised by higher pain levels and a greater metastatic burden, rather than treatment toxicity [22]. Although established thresholds offer guidance [16], they may overlook subtle yet clinically relevant changes, and HRQoL questionnaires should be regarded primarily as supportive instruments, with limitations in capturing the subjective nature of QoL [47], particularly in palliative settings. In this regard, disease-specific tools such as the FACT-P Symptom Index-17, may complement more general instruments such as FACT-P by providing a detailed evaluation of the symptom burden and QoL changes in patients with advanced or palliative-stage PC [48]. HRQoL data should inform, but not solely determine, clinical decision-making, with the individual patient context always taken into account. Overall, a more nuanced interpretation of HRQoL changes beyond predefined thresholds is essential for truly patient-centred decision-making.

The findings from our meta-analysis should be interpreted with caution. First, the analysis focused on patients with mild or no pain at baseline. Two of the three studies included explicitly enrolled only asymptomatic or mildly symptomatic patients (BPI-SF<4, no opioid use) [22,23]. The third reported a median baseline BPI-SF score of<4, suggesting that most participants probably had mild symptoms [25]. However, as the symptom level was not defined via explicit inclusion criteria, but rather inferred from the baseline characteristics reported, this introduces a degree of uncertainty and may have contributed to clinical heterogeneity in the pooled analysis. In addition, variation in the definitions of clinically meaningful change in HRQoL (thresholds of ≥6 vs ≥10 points on the FACT-P scale) across studies may introduce further heterogeneity into the interpretation of results. Notably, considerable statistical heterogeneity was observed across analyses, which probably reflects a combination of methodological and clinical differences between studies, including variations in trial design, baseline patient characteristics, and the timing and frequency of HRQoL assessments. Our meta-analysis revealed substantial heterogeneity across the studies included, which limits the reliability of the pooled estimates and underscores the need for future research with more homogeneous cohorts to better evaluate the impact of PARPi on HRQoL. Methodological differences were notable, particularly in study populations, as well as variations in follow-up duration and the timing of outcome assessments, all of which contribute to variability in the results. In addition, the inherently subjective and individual nature of QoL further complicates the interpretation of findings across this patient cohort. The lack of individual-level pain data further limits the generalisability to patients with more severe pain. Most of the data available are from subset analyses for RCTs, which are prone to inherent biases, including patient selection and post hoc modifications. Furthermore, variability in the timing of HRQoL assessments across trials may affect the comparability of outcomes. Potential publication bias cannot be excluded, as studies reporting significant HRQoL findings may be preferentially published. Moreover, HRQoL outcomes were primarily evaluated in patients with ECOG performance status ≤2, so the generalisability of the results is limited.

## 5 Conclusions

We found no statistically or clinically meaningful deterioration in HRQoL with PARPi treatment in patients with mCRPC, and one study suggested a potential benefit. This supports the favourable trade-off between oncological efficacy and tolerability, and underscores the patient-centred value of PARPi in the management of mCRPC. However, the current reliance on predefined numerical thresholds for FACT-P total scores may oversimplify the complex and individual experience of patients in the palliative mCRPC setting. With most of the differences observed falling below established thresholds for clinical meaningfulness, there are concerns that existing HRQoL instruments may not adequately reflect patient-relevant changes, particularly given the shift from a curative to a life-prolonging and symptom-alleviating therapeutic goal; even subtle variations may have substantial clinical and personal relevance in this setting and may differ from other settings.

  ***Author contributions***: Shahrokh F. Shariat had full access to all the data in the study and takes responsibility for the integrity of the data and the accuracy of the data analysis.

  *Study concept and design*: Roessler, Fazekas, Miszczyk.

*Acquisition of data*: Roessler, Matsukawa, Dematteis, Alfarhan, Alqahtani, Cormio.

*Analysis and interpretation of data*: Roessler, Miszczyk, Fazekas, Soeterik, Shariat.

*Drafting of the manuscript*: Roessler, Miszczyk.

*Critical revision of the manuscript for important intellectual content*: Miszczyk, Rajwa, Schuettfort, Vetterlein, Soeterik, Fazekas, Fisch, Leapman, Karakiewicz, Shariat.

*Statistical analysis*: Roessler, Miszczyk.

*Obtaining funding*: None.

*Administrative, technical, or material support*: None.

*Supervision*: Miszczyk, Fazekas, Shariat.

*Other*: None.

  ***Financial disclosures:*** Shahrokh F. Shariat certifies that all conflicts of interest, including specific financial interests and relationships and affiliations relevant to the subject matter or materials discussed in the manuscript (eg, employment/affiliation, grants or funding, consultancies, honoraria, stock ownership or options, expert testimony, royalties, or patents filed, received, or pending), are the following: Shahrokh F. Shariat reports honoraria form Astellas, AstraZeneca, BMS, Ferring, Ipsen, Janssen, MSD, Olympus, Pfizer, Roche, and Takeda; a consulting or advisory role for Astellas, AstraZeneca, BMS, Ferring, Ipsen, Janssen, MSD, Olympus, Pfizer, Pierre Fabre, Roche, and Takeda; and participation in speaker bureaus for Astellas, AstraZeneca, Bayer, BMS, Ferring, Ipsen, Janssen, MSD, Olympus, Pfizer, Richard Wolf, Roche, and Takeda. Margit Fisch reports honoraria from Astellas and Apogepha, and a grant from Boston Scientific. The remaining authors have nothing to disclose.

  ***Funding/Support and role of the sponsor*:** None.
