# Supplementary material for: Impact of PARP Inhibitors on Health-related Quality of Life in Patients with Metastatic Castration-resistant Prostate Cancer: A Systematic Review and Meta-analysis
Source: Eur Urol Open Sci. 2025 Sep 8;80:48–56. doi: 10.1016/j.euros.2025.08.008 (PMC12454867; doi:10.1016/j.euros.2025.08.008)

**Supplementary Figure 1 - Preferred Reporting Items for Systematic Reviews and Meta-analyses (PRISMA) - flow diagram for new systematic reviews which included searches of databases and registers only**

**Identification of studies via databases and registers**

Records excluded after duplicates removal

- (n = 57)

Records identified by database searching (n = 301):

MEDLINE: (n = 31)

Embase: (n = 219)

Web of Science (n = 51)

**Identification**

Records excluded during Title/Abstract screening

- (n = 230)

Records assessed for eligibility

(n = 244)

Reports sought for retrieval

(n = 14)

Reports not retrieved

- (n = 4)

**Screening**

Records excluded during full-text screening

- (n = 3)

Reports assessed for eligibility

(n = 10)

**Report included (n = 7)**

**Studies included (n = 7)**

**Included**

Source: Page MJ, et al. BMJ 2021;372:n71. doi: 10.1136/bmj.n71.

This work is licensed under CC BY 4.0. To view a copy of this license, visit <https://creativecommons.org/licenses/by/4.0/>

**Supplementary Table 1: Detailed Search Strategy for the databases**

MEDLINE (PubMed)

| # | Search string | Hits |
| --- | --- | --- |
| #1 | "prostatic neoplasms"[MeSH Terms] OR (prostat*[TIAB] AND (cancer[TIAB] OR carcinoma[TIAB] OR malignan*[TIAB] OR tumor[TIAB] OR tumour[TIAB] OR neoplas*[TIAB])) | 226.453 |
| #2 | "poly adp ribose polymerase inhibitors"[MeSH Terms] OR "PARP"[Title/Abstract] OR PARPIi[TIAB] OR olaparib[TIAB] OR rucaparib[TIAB] OR talazoparib[TIAB] OR veliparib[TIAB] OR niraparib[TIAB] OR pamiparib[TIAB] | 27.552 |
| #3 | "quality of life"[MeSH Terms] OR "quality of life"[Title/Abstract] OR "QOL"[Title/Abstract] OR "HR-QoL"[Title/Abstract] OR "HRQoL"[Title/Abstract] OR "patient-reported outcomes"[Title/Abstract] OR "pain progression"[Title/Abstract] | 519.466 |
| #4 | #1 #2 #3 | 31 |

Embase

| # | Search string | Hits |
| --- | --- | --- |
| #1 | (prostatic neoplasm'/exp OR (prostat*:ti,ab AND (cancer:ti,ab OR carcinoma:ti,ab OR malignan*:ti,ab OR tumor:ti,ab OR tumour:ti,ab OR neoplas*:ti,ab))) AND ('poly adp ribose polymerase inhibitor'/exp OR PARP:ti,ab OR PARPi:ti,ab OR olaparib:ti,ab OR rucaparib:ti,ab OR talazoparib:ti,ab OR veliparib:ti,ab OR niraparib:ti,ab OR pamiparib:ti,ab)  AND ('quality of life'/exp OR 'quality of life':ti,ab OR QOL:ti,ab OR HR-QoL:ti,ab OR HRQoL:ti,ab OR 'patient-reported outcomes':ti,ab OR 'pain progression':ti,ab) | 219 |

Web of Science

| # | Search string | Hits |
| --- | --- | --- |
| #1 | TS=(prostat* AND (cancer OR carcinoma OR malignan* OR tumor OR tumour OR neoplas*)) AND TS=("poly adp ribose polymerase inhibitor*" OR PARP OR PARPi OR olaparib OR rucaparib OR talazoparib OR veliparib OR niraparib OR pamiparib) AND TS=("quality of life" OR QOL OR HR-QoL OR HRQoL OR "patient-reported outcomes" OR "pain progression") | 51 |

**Supplementary Figure 2: PICOS framework (population, intervention, comparison, outcome, and study design)**

- P: Patients with metastatic prostate cancer (hormone sensitive, castration resistant)
- I: Treatment with Poly(ADP-ribose) polymerase inhibitor (PARPi) agents +/- additional systemic therapy
- C: Patients assigned to different treatment methods than PARPi – outcomes from single Arm studies will also be pooled to assess health related quality of life (hQoL) response rates
- O: HRQoL (e.g. EQ-5D-5L; FACT-P)
- S: prospective trials, including subset and post-hoc analyses of the study groups

**Supplementary Table 2: Risk of bias domains according to RoB2 - tool and ROBIS-I - tool of the included studies**

| **Study** | **Bias due to randomisation** | **Bias due to deviation from the intended intervention** | **Bias due to missing data** | **Bias due to outcome measurement** | **Bias due to selection of the reported results** |
| --- | --- | --- | --- | --- | --- |
| Clarke, N. W., et al. (2025) | As a post hoc analysis, there are no concerns about bias due to randomization.  Low Concerns | As a post hoc analysis, there are no concerns about bias due to deviation from the intended intervention.  Low Concerns | There are some concerns regarding bias due to missing data, as data from all patients were not collected  Some Concerns | There are low concerns regarding bias due to outcome measurement  Low Concerns | There are high concerns regarding bias due to the selection of reported results, as patients were split into asymptomatic and symptomatic groups, introducing bias in HRQoL  High Concerns |
| Rathkopf, D. E., et al. (2024) | As a post hoc analysis, there are no concerns about bias due to randomization.  Low Concerns | As a post hoc analysis, there are no concerns about bias due to deviation from the intended intervention.  Low Concerns | There are some concerns regarding bias due to missing data, as data from all patients were not collected  Some Concerns | There are low concerns regarding bias due to outcome measurement  Low Concerns | There are low concerns regarding bias due to selection of the reported results  Low Concerns |
| Saad, F., et al. (2022) | As a post hoc analysis, there are no concerns about bias due to randomization.  Low Concerns | There are some concerns about bias due to deviation from the intended intervention (phase-2 study)  Some Concerns | There are some concerns regarding bias due to missing data, as data from all patients were not collected  Some Concerns | There are low concerns regarding bias due to outcome measurement  Low Concerns | There are low concerns regarding bias due to selection of the reported results  Low Concerns |
| Thiery-Vuillemin, A., et al. (2022) | As a post hoc analysis, there are no concerns about bias due to randomization.  Low Concerns | As a post hoc analysis, there are no concerns about bias due to deviation from the intended intervention.  Low Concerns | There are some concerns regarding bias due to missing data, as the FACT-P was not assessed as mean change from baseline and data from all patients were not collected  Some Concerns | There are low concerns regarding bias due to outcome measurement  Low Concerns | There are low concerns regarding bias due to selection of the reported results  Low Concerns |
| Matsubara, N., et al. (2025) | As a post hoc analysis, there are no concerns about bias due to randomization.  Low Concerns | As a post hoc analysis, there are no concerns about bias due to deviation from the intended intervention.  Low Concerns | There are some concerns regarding bias due to missing data, as the FACT-P was not assessed as mean change from baseline and data from all patients were not collected  Some Concerns | There are low concerns regarding bias due to outcome measurement  Low Concerns | There are low concerns regarding bias due to selection of the reported results  Low Concerns |


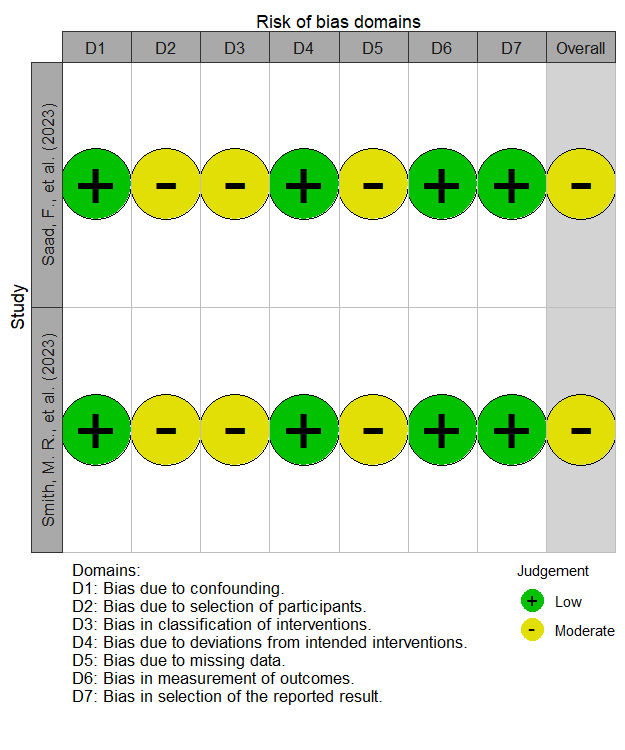

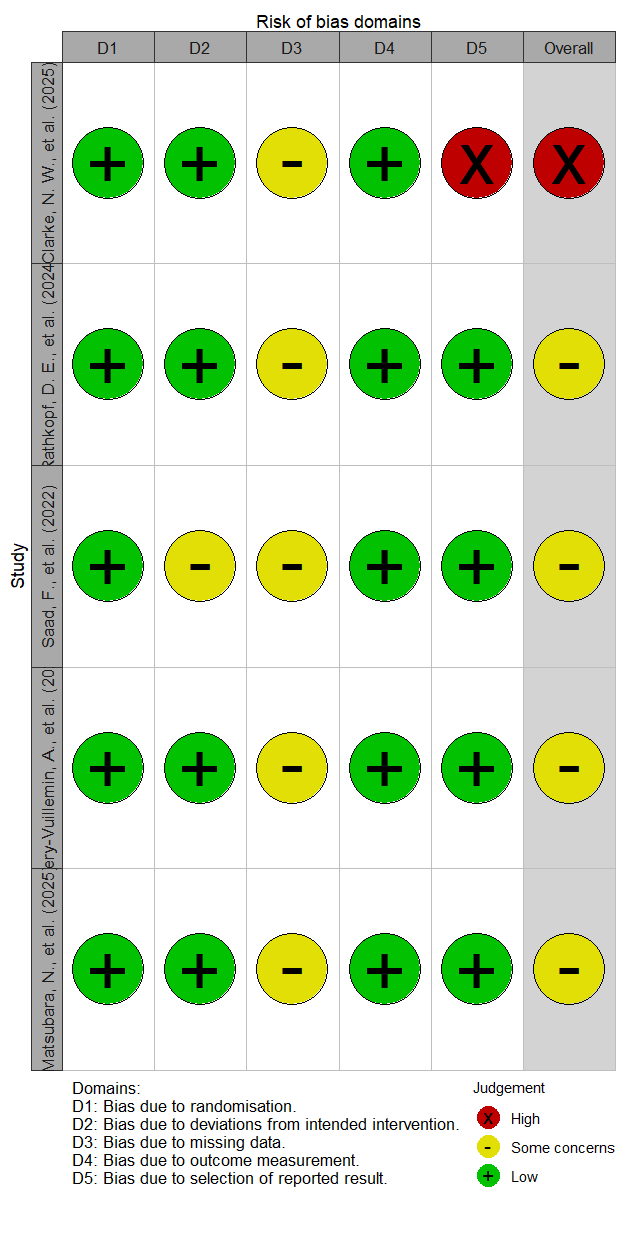


**Supplementary Table 3 – Characteristics of the included studies**

| Study | Clinical trial name | Clinical trial phase | Trial period | Assessment of questionnaires (EQ-5D-5L and FACT-P) |
| --- | --- | --- | --- | --- |
| Clarke, N. W., et al. (2025) | PROpel | Phase-3 | 2018-2020 | weeks 1, 3, 5, 7, 9, 11, and 13, followed by every 4 weeks starting from week 13 |
| Rathkopf, D. E., et al. (2024) | MAGNITUDE | Phase-3 | 2019-2023 | baseline, on the first day of designated treatment cycles, and then every 3 months for up to 2 years after the end of treatment |
| Saad, F., et al. (2023) | TALAPRO-1 | Phase-2 | 2017-2020 | baseline, every 2 weeks until week 9, every 4 weeks until week 25, and then every 12 weeks until disease progression |
| Matsubara, N., et al. (2025) | TALAPRO-2 | Phase-3 | 2019-2020 | baseline, and at scheduled visits (every 4 weeks until week 53, then every 8 weeks) until centrally determined radiographic progression |
| Saad, F., et al. (2022) | NCT01972217 | Phase-2 | 2014-2015 | baseline, weeks 4, 8, and 12, and then every 12 weeks until treatment discontinuation |
| Smith, M. R., et al. (2023) | GALAHAD | Phase-2 | 2016-2020 | baseline, on day 1 of cycles (28 days) 3, 5, and 7, and then on day 1 of every third cycle until treatment completion. |
| Thiery-Vuillemin, A., et al. (2022) | PROfound | Phase-3 | 2017-2019 | baseline and then every 8 weeks until 24 weeks after progression or discontinuation of randomised treatment |

**Supplementary Figure 3 – Leave-one-out analysis of the included studies in the meta-analysis**

Early**
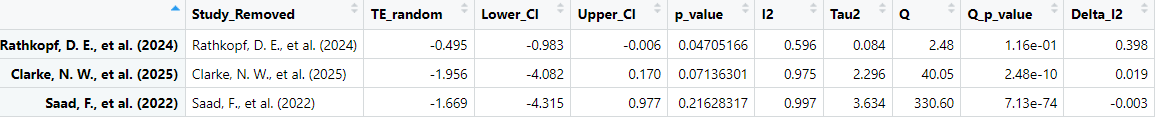
**


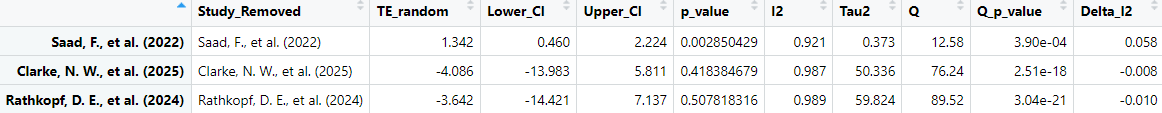
Late

**Supplementary Figure 4 – Forest plot: Pooled mean differences of FACT-P total scores for treatment- and control arm in early and late time period after excluding source of heterogeneity**


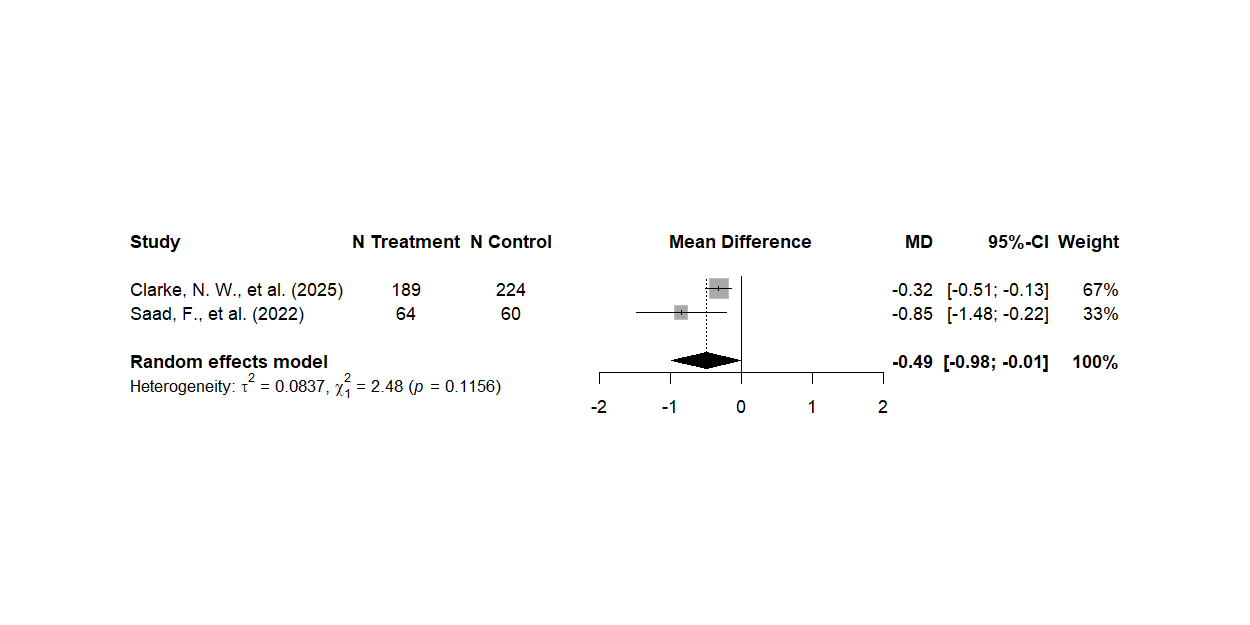
Early


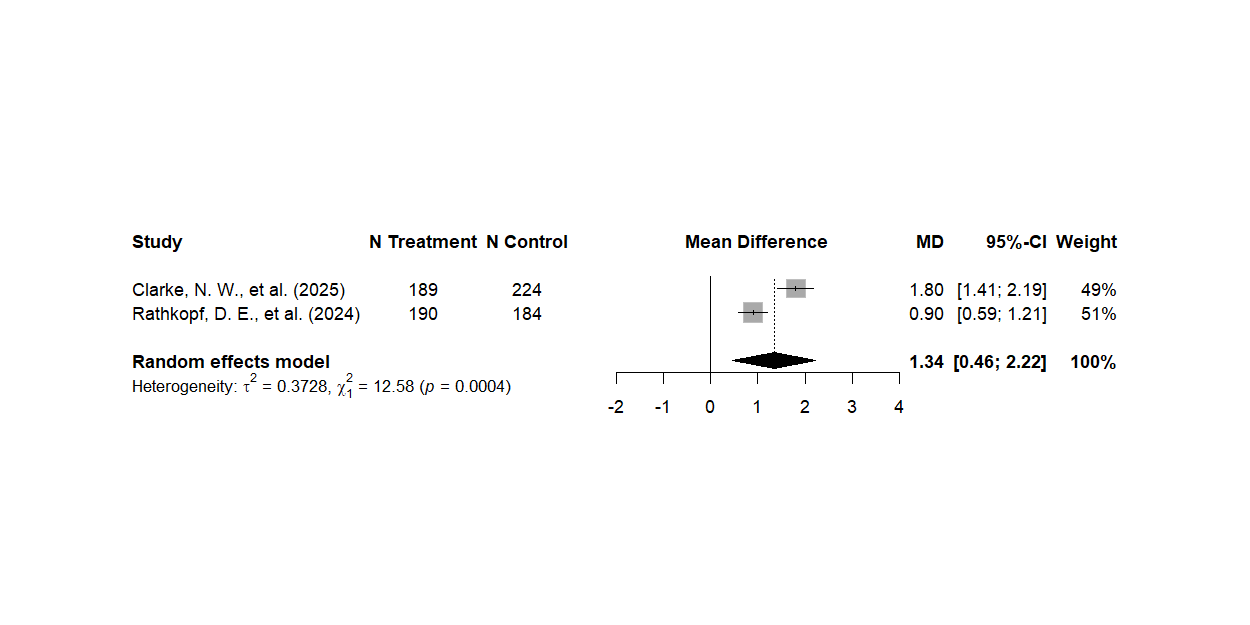
Late

**Supplementary File 8: AMSTAR2 – Checklist**


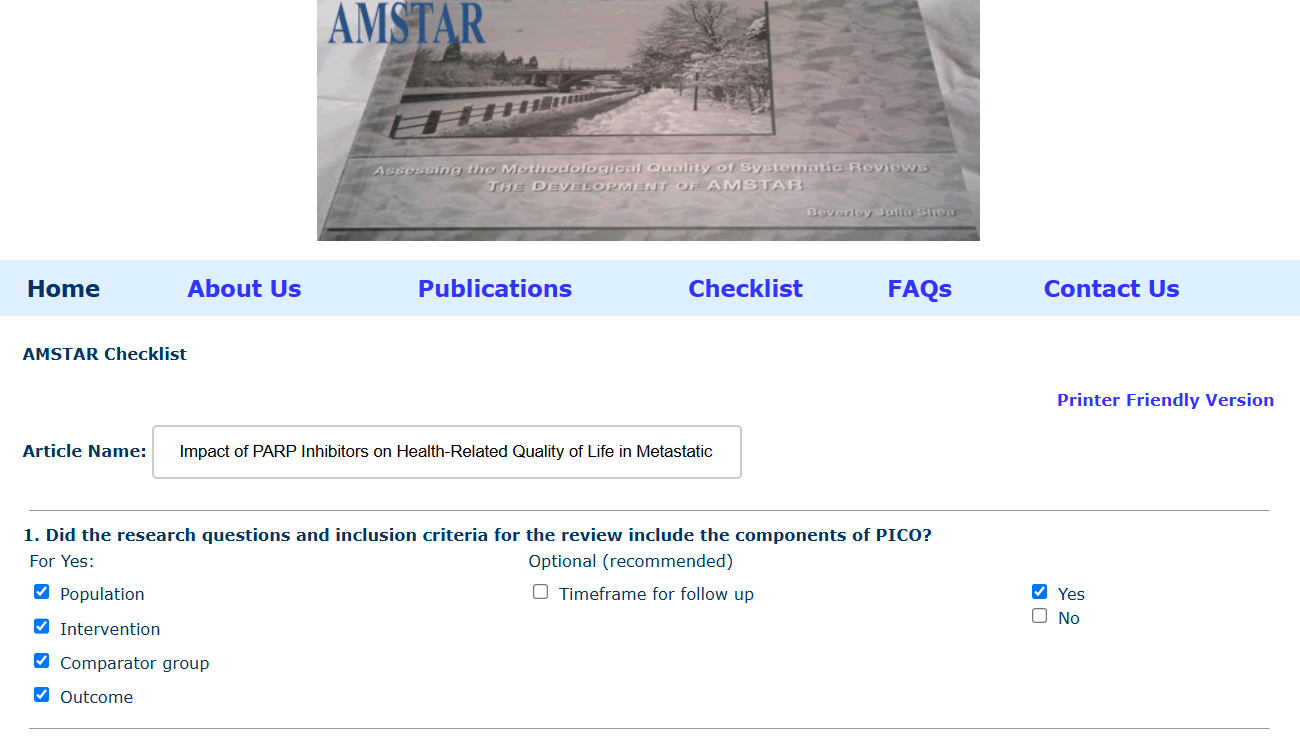

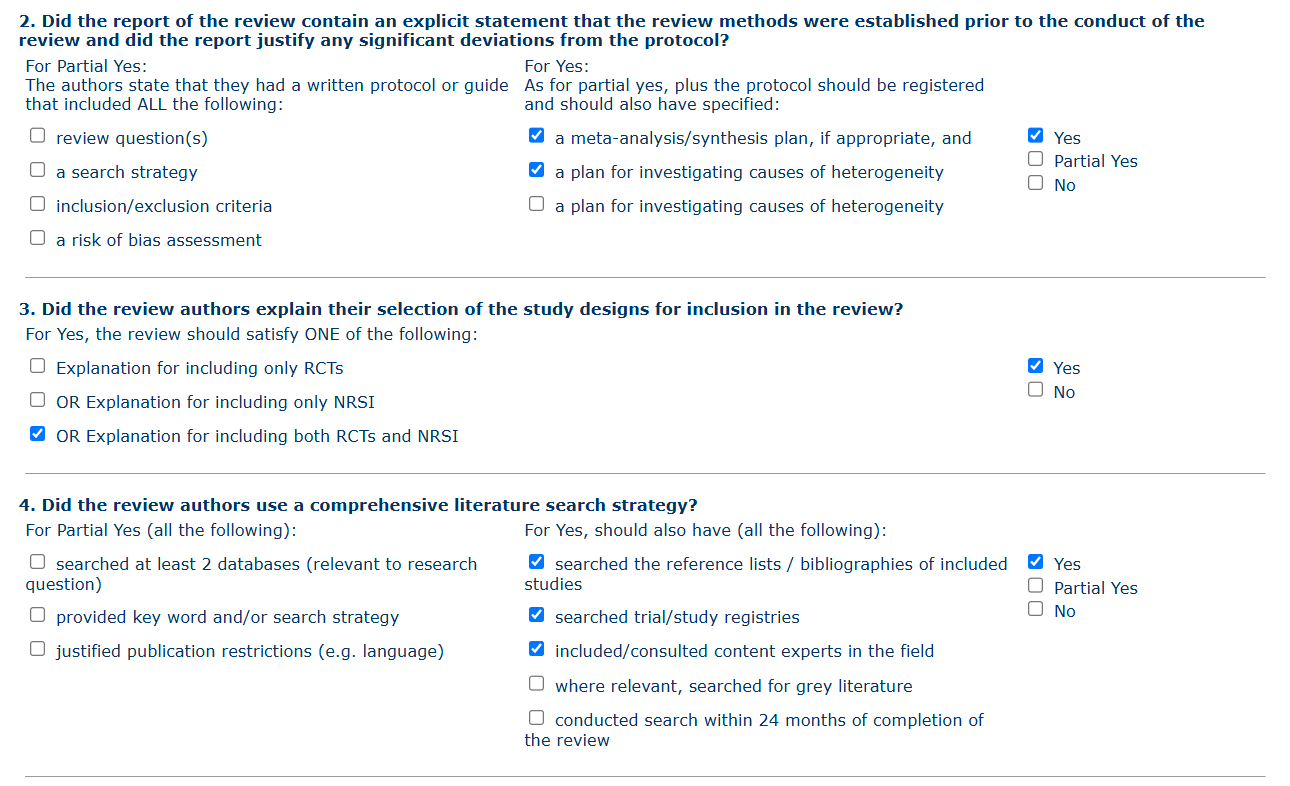

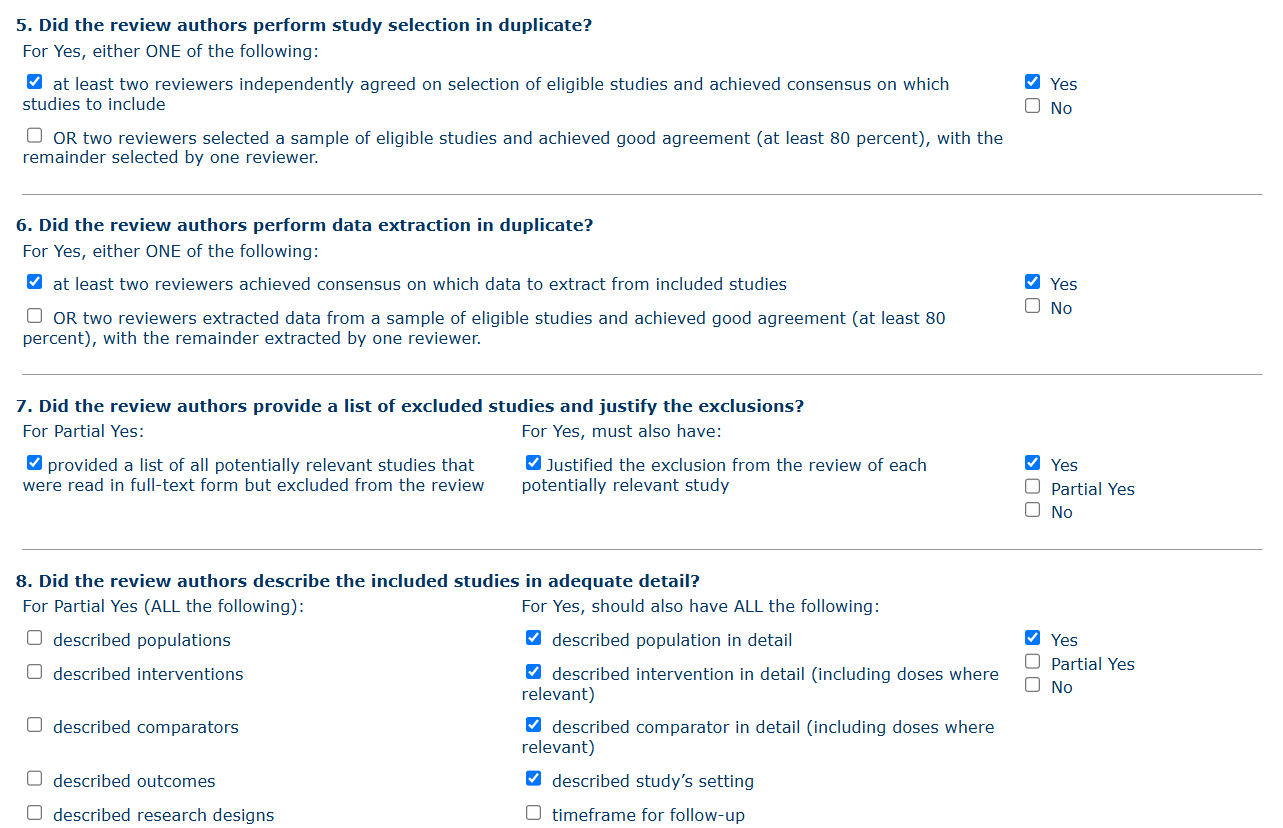

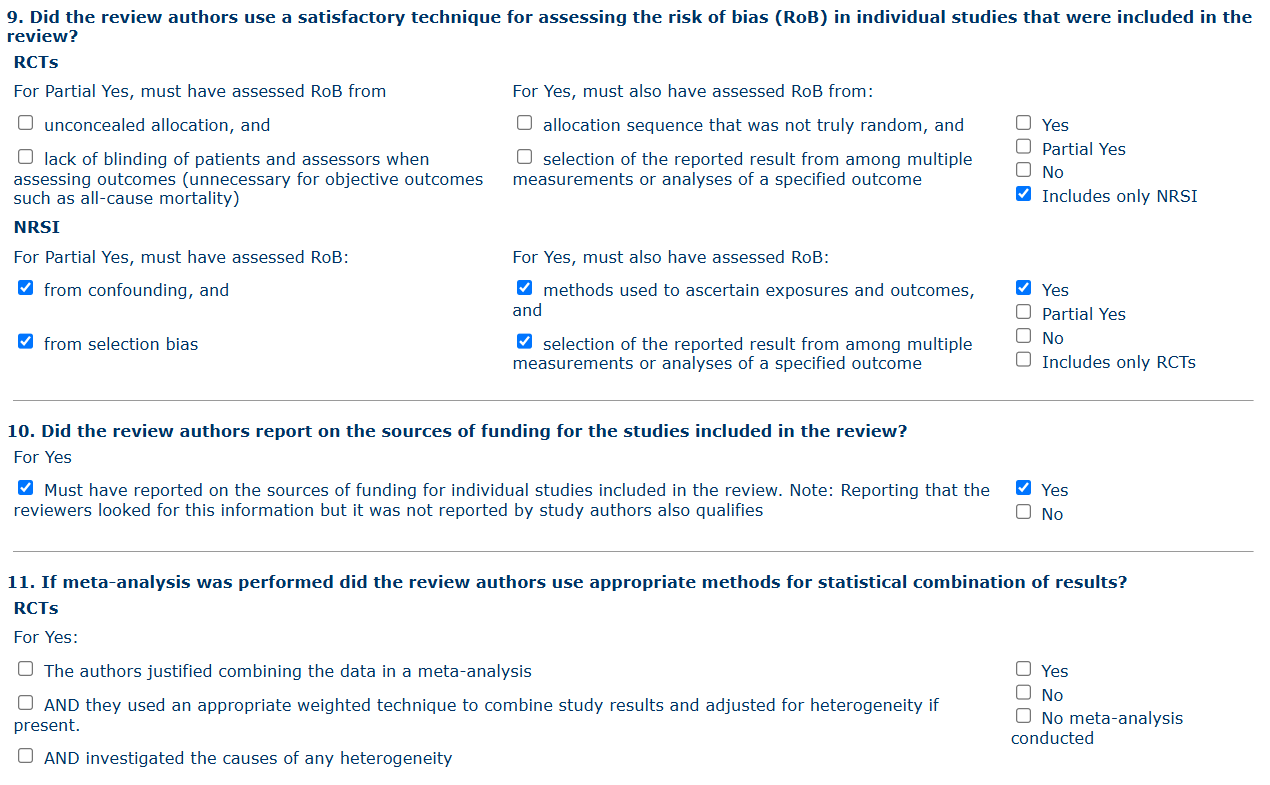

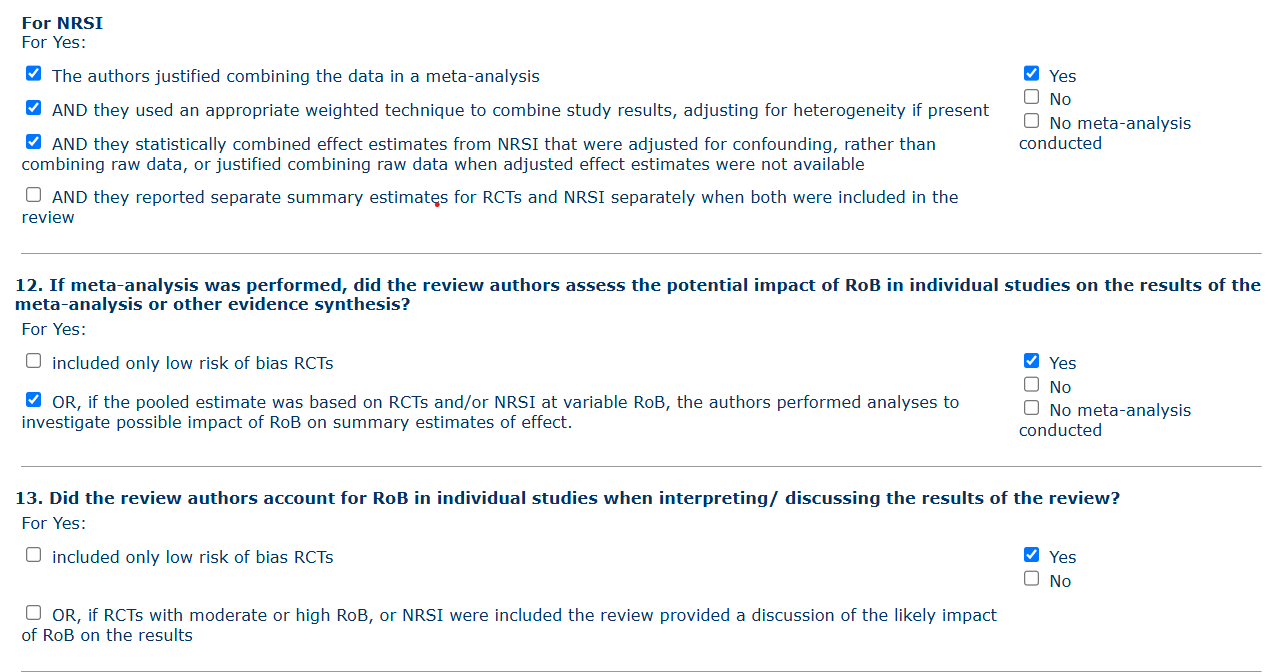

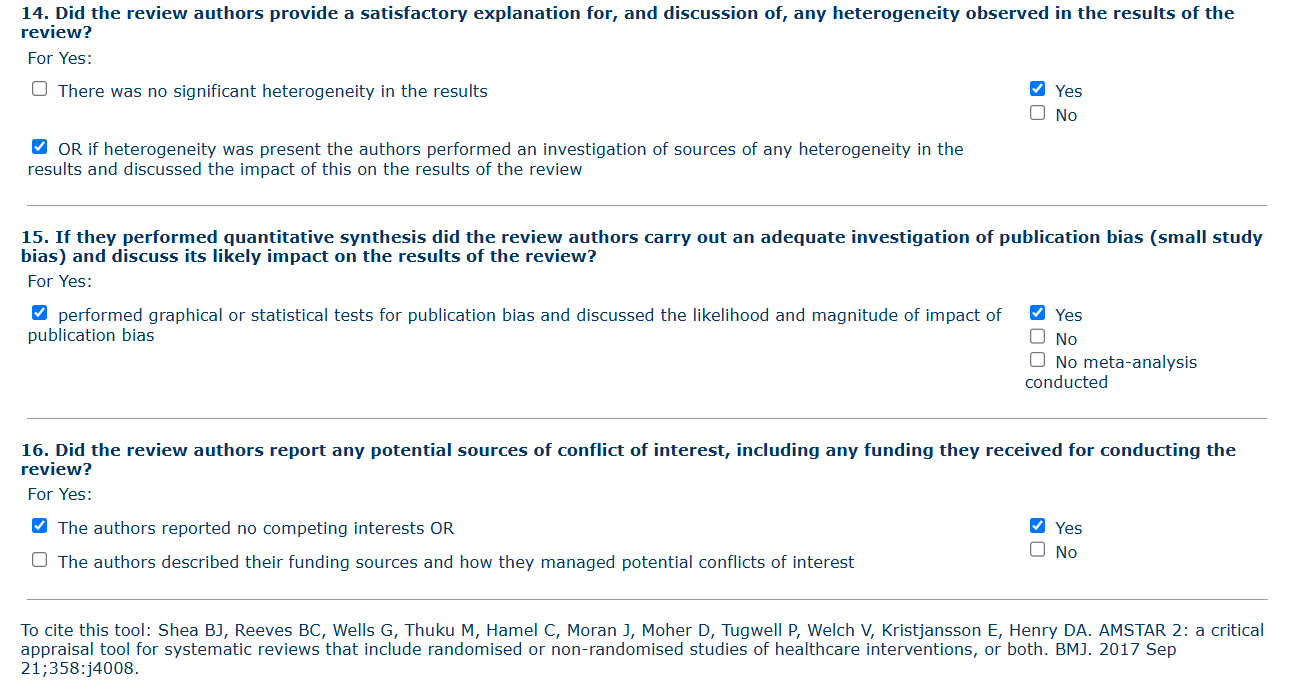

Supplement: Supplementary Data 1 [file mmc1.docx]
